# Questionnaire-based Evaluation of Factors Leading to Patient-physician Distrust and Violence against Healthcare Workers

**DOI:** 10.5005/jp-journals-10071-23203

**Published:** 2019-07

**Authors:** Shruti Sharma, Parshotam Lal Gautam, Sarit Sharma, Amandeep Kaur, Neha Bhatia, Gurpreet Singh, Paawanjot Kaur, Anhad Kumar

**Affiliations:** 1,2,4,5 Department of Critical Care Medicine, Dayanand Medical College and Hospital, Ludhiana, Punjab, India; 3 Department of Community Medicine, Dayanand Medical College and Hospital, Ludhiana, Punjab, India; 6,7,8 Dayanand Medical College and Hospital, Ludhiana, Punjab, India

**Keywords:** Communication, Healthcare workers (HCWs), Patient physician distrust, Workplace Violence (WPV)

## Abstract

**Background:**

Rising incidents of aggression and violence against healthcare workers (HCWs) is widening the rift between the patients and their caregivers. So, aim of the study was to evaluate the perceptions of healthcare workers (HCWs) and patient's attendants about factors responsible for widespread violence and patient-physician distrust.

**Materials and methods:**

An anonymous, questionnaire-based, cross-sectional study was conducted over a period of one year in a tertiary care teaching institute. Performas, adapted from WHO published questionnaire and translated to local language, were administered separately to HCWs and attendants. Responses generated were collected and analyzed.

**Results:**

Out of 295 HCWs, 11 (3.7%) HCWs faced physical violence, whereas verbal abuse was faced by 147 (50%) HCWs. A higher number of incidents of physical violence (91%) and verbal abuse (64%) were faced by HCWs in the age group of 20-30 years. Verbal abuse was faced by 49.3% of nurses, 53% of junior residents, 61% of senior residents and 36% of consultants. Out of 158 incidents of workplace violence (WPV), maximum occurred in ICUs (62.0%) and emergency (21%). Unexpected death, unexpected complication, extended hospital stay, staff shortage and unexpected bill were some of the factors perceived to be responsible for WPV.

**Conclusion:**

HCWs commonly face violence from patient's attendants resulting in stressful and fearful environment at the healthcare facility. Dedicated sessions on good communication and counseling for HCWs and better security arrangements at the hospitals are the need of the hour and also in the best interest of HCWs and patients.

**How to cite this article:**

Sharma S, Gautam PL, Sharma S, Kaur A, Bhatia N, Singh G, *et al*. Questionnaire-based Evaluation of Factors Leading to Patient-physician Distrust and Violence against Healthcare Workers. Indian J Crit Care Med 2019;23(7):302-309.

## INTRODUCTION

Patient-physician trust has been the basis of medical practice since ancient times. In the recent years rising incidents of aggression and violence against healthcare workers (HCWs) reflect widening rift between the patients and their caregivers. Workplace violence in healthcare is endemic worldwide and currently it has emerged as an Indian healthcare crisis. In USA, the rate of assaults on HCWs is higher than that of other jobs reported as 8 assaults per 10000 workers compared with 2 per 10000 for the general workplace.^1^

According to a World Health Organization (WHO) report, between 8% and 38% of HCWs suffer physical violence at some point in their career.^2^ Workplace Violence (WPV) can affect the physical and mental health of victims affecting productivity. It results in degradation of the work environment and morale of HCWs. It is believed that 70% to 80% of assaults are not reported.^1^ In India also HCWs face threats and physical violence from irate relatives. We have moved from ‘Doctor, Please Save me’ to ‘Please Save the doctor’ and it is time to reflect what has gone wrong. According to a study by the Indian Medical Association, more than 75% of doctors have faced violence at work.^3^ Many hospitals have been ransacked by emotional relatives of patients as an aftermath of sudden unexpected deaths.^4–7^ The problem of WPV is multifactorial in origin. Triggering factors may be demise of the patient, inflated bills, poor infrastructure, and behavior of the HCWs etc. So, the present study was conducted to know the prevalence of violence among the HCWs, and to evaluate the perceptions of HCWs and patient's attendants about various factors responsible for patient-physician distrust and violence against HCWs.

## MATERIALS AND METHODS

### Study Setting

This study was conducted in the Medical, Surgical and Neurosurgery ICUs of a tertiary care teaching institute in North India after approval by Institutional Ethics Committee. The primary treating physicians/surgeons take rounds in the ICUs and interact with the patients and attendants. Patients are also managed by a team of intensivists and post graduate residents from related specialties round the clock. Interns under training are also posted in each ICU during the day shift.

### Study Design

This was an anonymous, questionnaire-based, cross-sectional study conducted over a period of one year from August 2017 to July 2018. Respondents’ name and identity were kept confidential. Study participants were HCWs including doctors and staff nurses posted in the ICUs, and doctors visiting the ICUs for daily rounds. Responses of attendants of the patients admitted to ICUs during the study period were also recorded on another questionnaire-based performa.

## DEFINITIONS^8^

### Workplace Violence (WPV)

Incidents where staff is abused, threatened or assaulted in circumstances related to their work, including commuting to and from work, involving an explicit or implicit challenge to their safety, well-being or health. Violence includes physical assault, homicide, verbal abuse, bullying/mobbing, sexual and racial harassment and psychological stress.

### Physical Violence

The use of physical force against another person or group that results in physical, sexual or psychological harm. It includes beating, kicking, slapping, stabbing, shooting, pushing, biting, and pinching, among others.

### Psychological Violence (Emotional Abuse)

Intentional use of power, including threat of physical force, against another person or group, that can result in harm to physical, mental, spiritual, moral or social development. It includes verbal abuse, bullying/mobbing, harassment, and threats. For purpose of simplification in the present study, WPV was categorized in to two categories: Physical Violence and Verbal Abuse.

### Performa

The performas were adapted and modified from survey questionnaire provided by WHO and were developed in English, Hindi and Punjabi as these were the commonly spoken and written languages in our state.^8^ The performas were validated for comprehension, ease of understanding and to eliminate language barriers among HCWs and attendants of patients. Performa A had 28 items and was administered to HCWs (doctors and staff nurses) and information regarding demography, profession, designation, work experience, shift duty and recollection of physical or verbal abuse in past one year was collected. Performa B had 20 items and was administered to patient's attendants and included questions about demography, education and awareness about rising incidents of violence against HCWs. Relatives were also asked about their involvement in any incident of WPV. At the end of both the performas, HCWs and attendants of patients were asked about their perceptions about the factors responsible for WPV as well as suggestions to improve patient physician relationship.

## DATA COLLECTION

Performa A was distributed to HCWs (doctors and nurses) posted in the ICUs as well as to doctors during their visits in the ICUs. Performas were collected after 24 hours. The interns posted in each ICU were trained in administering the performa B to the patients’ attendants and assisting them in filling it. Their progress was supervised by the principal investigator from time to time. Interns administering the performas were not involved in interpretation of results.

## DATA ANALYSIS

The data generated was entered into Microsoft excel. The quantitative analysis of the data was done by calculating percentages and chi square test using Statistical Package for the Social Sciences (IBM SPSS Statistics for Windows, Version 20.0. Armonk, NY: IBM Corp.). P value of <0.05 was taken as significant. The qualitative analysis was conducted by grouping the similar response to the open ended questions to bring out important suggestions and themes to know the factors responsible for patient physician distrust.

## RESULTS

**Fig. 1:**
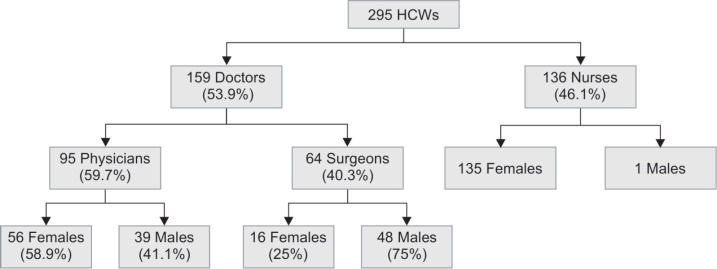
Distribution of participants according to professional group and gender

A total of 308 performas were distributed to HCWs out of which 295 completed performas were collected generating a response rate of 96% approximately. Study participants included 159 doctors (95 physicians, 64 surgeons), 136 nurses (Fig. 1) and 142 attendants of patients. Among doctors, 36 consultants, 23 senior residents and 100 junior residents were part of the study. Mean age distribution of the HCWs was 30.74±6.89, 32.30±8.76 and 30.13±5.17 for physicians, surgeons and nurses respectively.

## HEALTHCARE WORKERS

**Fig. 2:**
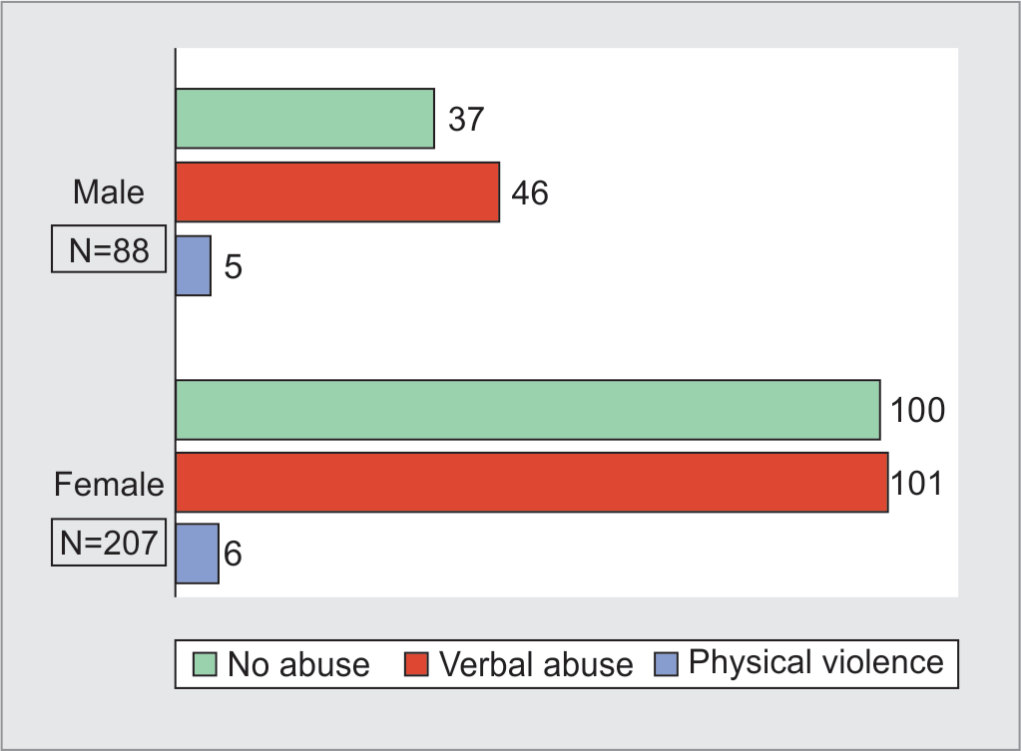
Distribution of workplace violence with respect to gender

**Fig. 3:**
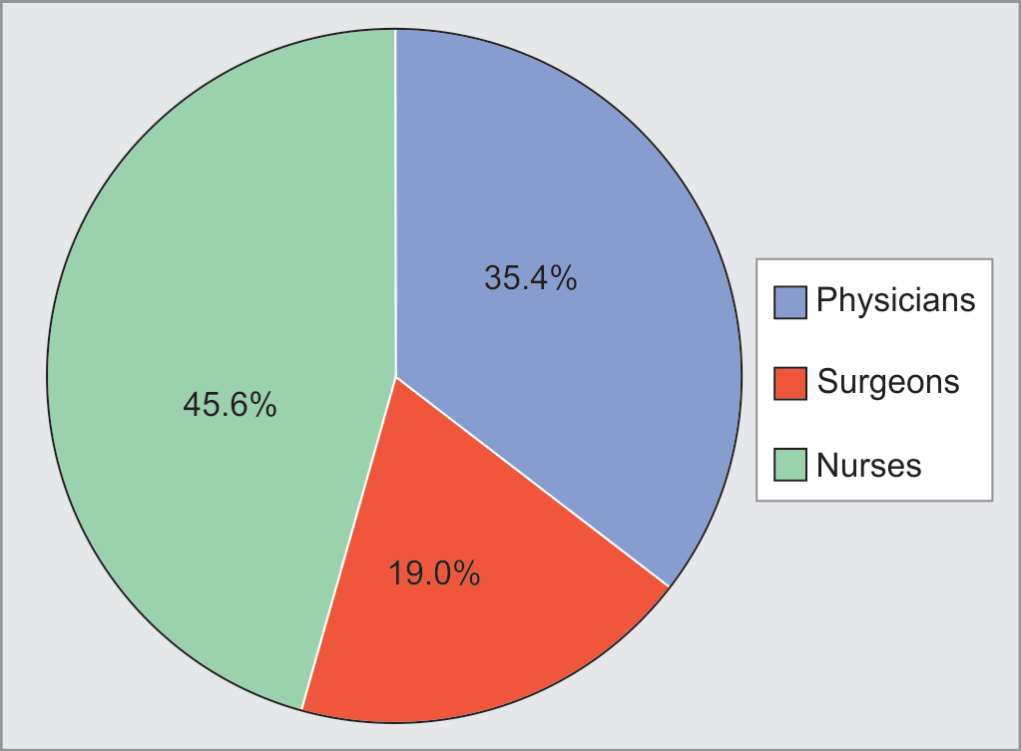
Distribution of verbal abuse with respect to profession

**Fig. 4:**
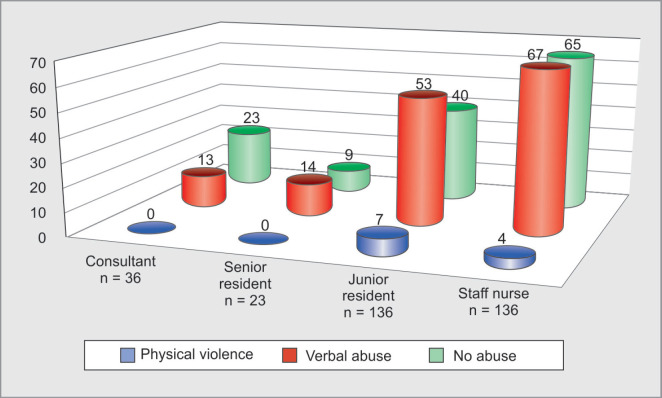
Distribution of workplace violence with respect to designation

Out of 295 HCWs, 158(54%) reportedly suffered WPV. Physical violence was faced by 11(3.7%) HCWs, whereas verbal abuse was faced by 147 (50%) HCWs during the past one year. A higher number of incidents of physical violence (91%) and verbal abuse (64%) were faced by HCWs in the age group of 20-30 years (Table 1). Distribution of verbal abuse with respect to age of HCWs is statistically highly significant (p value=0.004). Physical violence was experienced by 6 female and 5 male HCWs, whereas 101 (68.7%) female and 46 (31.3%) male HCWs faced verbal abuse (Fig. 2). Physical violence was faced by 4 physicians, 3 surgeons and 4 nurses as shown in Table 1. Distribution of verbal abuse with respect to profession in our study was 45.6% by nurses, 35.4% by physicians and 19.0% by surgeons (Fig. 3). As seen in Table 2, among the HCWs who faced physical violence, 10 (90.9%) were of less than 5 years’ experience, whereas those who faced verbal abuse 92 (62.6%) participants were of experience less than 5 years and the relationship between years of experience and verbal abuse was statistically significant (p value=0.025). On comparing WPV with respect to designation we found that 7 junior residents and 4 nurses faced physical violence, whereas 67(45.6%) nurses, 53 (36.1%) junior residents, 14 (9.5%) senior residents and 13(8.8%) consultants reported to have faced verbal abuse (Fig. 4). On intra-group analysis, 49.3% of nurses and 53% of junior residents, 61% of senior residents and 36% of consultants experienced verbal abuse.

**Table 1:** Prevalence of workplace violence with respect to age and profession

	*Parameter*	*N*	*Physical violence*	*Verbal abuse*
**Age in years**	20–30	176 (59.7)	10 (90.9)	94 (64)
	30–40	83 (28.1)	1 (9.1)	45 (30.7)
	40–50	31 (10.5)	0	6 (4)
	50–60	5 (1.7)	0	2 (1.3)
	Total	295	11	147
	P value		0.06 (d.f.=2)	0.004 (d.f.=3) HS
**Profession**	Physicians	95 (32.2)	4 (36.4)	52 (35.4)
	Surgeons	64 (21.7)	3 (27.2)	28 (19.0)
	Nurses	136 (46.1)	4 (36.4)	67 (45.6)
	Total	295	11	147
	*P* value		0.794	0.391

Figures in parentheses indicate percentages

Among study participants, 135 nurses (99%), 73 junior residents (73%), 10 senior residents (43%) and 8 consultants (22.2%) did shift duty. HCWs on shift duty reported higher incidence of physical violence (81.8%) and verbal abuse (78.9%) than those who do not do shift duty (Table 2). It was found that all the 11 physical assaults and 106 (72%) incidents of verbal abuse occurred in HCWs associated with night shift (8.00P.M and 8.00 A.M) or night on call.

**Fig. 5:**
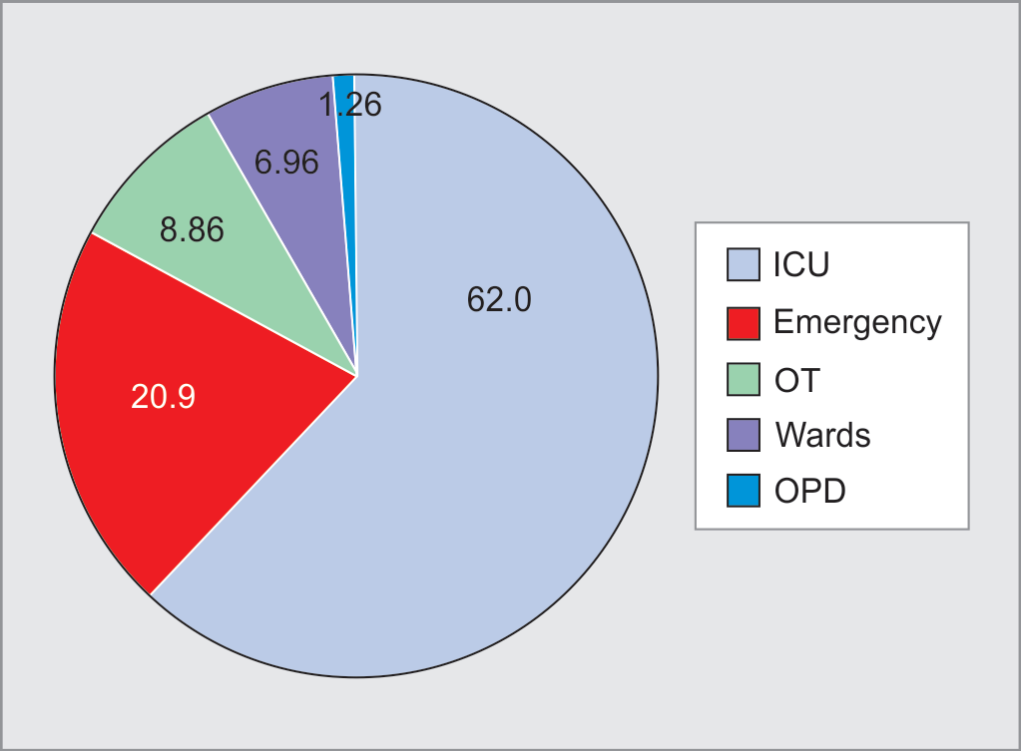
Distribution of workplace violence with respect to location

In 97% of incidents, assailant was attendant of the patient except in 5 incidents where it was the patient himself. All the incidents of WPV occurred inside the hospital. Incidences of verbal abuse were reported to be most frequent from 8.00pm to 8.00 am (72%) followed by that between 2.00 pm and 08.00pm (22%), whereas all the incidents of physical violence were reported to be between 8.00 pm to 8.00 am (night shift). Five incidents of physical violence occurred in the emergency and six in the ICUs. Out of 158 incidents of WPV faced by HCWs, 98 (62.0%) occurred in ICUs, 33 (20.9%) in emergency, 14 (8.86%) in operation theatre, 11(6.96%) in wards and 2(1.26%) in OPD in the past one year (Fig. 5).

Frequency of facing WPV was reported as once by 46 (29%), 2-4 times by 54 (34%), 5-10 times by 41(26%) and several times by 17(11%) HCWs. In response to these incidents of WPV, counseling of attendants was done in 114 (72%) incidents, reporting to higher authorities was done in 77(49%) incidents, no reaction and continuation of treatment was done in 36 (23%) incidents, backfiring by HCWs physically or verbally in 13(8.2%) incidents, verbal warning was given to relatives in 29(18.3%) incidents and in only one incident police report was made and legal action taken.

**Table 2:** Prevalence of workplace violence with respect to experience, designation and shift timings

	*Parameter*	*N*	*Physical violence*	*Verbal abuse*
**Experience**	≤ 5yrs	172 (58.3)	10 (90.9)	92 (62.6)
	6–10	60 (20.3)	1 (9.1)	26 ((17.7)
	11–15	45 (15.3)	0	26 (17.7)
	16–20	14 (4.7)	0	2 (1.4)
	>20 yrs	4 (1.4)	0	1 (0.7)
	Total	295	11	147
	*p* value		0.264	0.025 (S)
**Designation**	Consultant	36 (12.2)	0	13 (8.8)
	Senior resident	23 (7.8)	0	14 (9.5)
	Junior resident	100 (33.9)	7 (63.6)	53 (36.1)
	Staff nurse	136 (46.1)	4 (36.4)	67 (45.6)
	Total	295	11	147
	*p* value		0.139	0.236
**Shift duty**	Yes	226	9 (81.8)	116 (78.9)
	No	69	2 (18.2)	31 (21.1)
	Total	295	11	147
	*p* value		0.678	0.352

Figures in parentheses indicate percentages

Among the HCWs who faced physical violence and verbal abuse, responses were also elicited about stress experienced by them (Table 3). Moderately to extremely disturbing memories due to incident of WPV were reported by 39(24.7%) HCWs whereas about 50 HCWs (31.6%) had the feelings of moderate to extreme avoidance response to the talking or thinking about the incident. One hundred nineteen (75.3%) participants felt moderately to extremely super-alert at all the times, 94 (59.4%) participants felt moderately to extremely burdened and felt everything they did was an effort while 17 HCWs took time off from work (1-7 days) due to high levels of stress.

Perceptions of the HCWs about the factors responsible for WPV were noted (Table 4). Among the 25 responses generated for factors responsible for physical violence and 367 responses generated for verbal abuse, unexpected death, unexpected complication, patient unlikely to improve, extended hospital stay, staff shortage, poor hospital administration, stress about patient's condition, unexpected bill and political links of patients or attendants were some of the important factors as perceived by HCWs (Table 4).

Suggestions given by HCWs to improve patient physician relationship were also noted and similar answers were grouped together and tabulated. Suggestions were categorized into administrative, HCWs and attendants related suggestions so as to have a clear understanding (Table 5).

Frequent counselling sessions, explaining clinical condition and plan of care in detail, improved nurse/doctor patient ratio, mutual respect and teamwork and strict and proactive security services were some of the suggestions given by HCWs to improve patient physician relationship and decrease WPV.

Some of the suggestions verbatim to improve patient physician relationship given by HCWs were:

- Need for training/workshops on communication skills between residents and staff with patients and attendants.
  Before start of residency doctor needs to be properly evaluated to see if there is any personality disorder or anger management issues.    Physician
- Educate people about medical stream that disease is not a doctor's fault but is with what patients presents when coming to the hospital and complications are also not doctor's fault so stop blaming the HCW for everything. Limit attendants and increase personnel. Invest more in healthcare system.    Surgeon
- Increase manpower. Counseling should be provided to attendants about patient's rights and employees’ rights. Attendants should be counseled to cooperate with the healthcare team.    Nurse

### Patient's Attendants

Filled performas from the 142 attendants of patients in the ICUs during the period of study were also collected and analyzed. Eighty four (59%) attendants were in the age group of 20-40 years, 48(34%) were females and 105(74%) attendants were married. Eighty four (59%) attendants had a rural background. The attendants’ study population comprised of 34 housewives, 30 government/private job holders, 25 farmers, 24 businessmen, 17 students, 9 laborers, and 3 unemployed persons on the basis of occupation. Forty-five attendants were educated up to matric, 33 were graduate, 28 were educated up to high school, 21 were postgraduate and 15 attendants were illiterate.

Awareness about increasing incidence of violence against HCWs was present in 88 (62%) patients’ attendants. Awareness was present in 24 (80%) Govt and Private job holders, 13 (76.4%) students, 15 (62.5%) businessmen, 18 (53%) housewives, and 13 (52%) farmers. Awareness about rising incidents of violence against HCWs was reported in 22 (79%) of attendants with high school education, 25 (76%) of graduates, 16 (76%) in post graduate attendants, 19 (42%) matric holders, and 6 (40%) in illiterate population. Awareness was present in 60 (63.8%) male attendants and 28 (58.3%) female attendants. Four attendants reported to have been involved in physical confrontations and they were of age between 20-30 years old students. Seventeen attendants reported having a verbal confrontation with the HCWs in the past one year. Eight attendants involved in verbal abuse were of 20-30 years age group and 7 attendants were of age 30-40 years and 2 were of 40-50 years age group. Out of these 21 incidents of WPV, in 13 incidents nurses were the victims whereas in 8 incidents doctors were the victims as reported by the attendants. All the incidents took place inside the hospital during past one year. Counseling of attendants was done by HCW/senior doctor/administrator in 12 incidents, no action and continuation of treatment in 5 cases, backfiring physically and verbally in 4 incidents, verbal warning in 2 incidents was given as reported by the attendants.

Six attendants who were involved in these incidents perceived that doctors’ behaviour was not upto the mark. The reasons for the WPV as stated by the attendants were unexplained complication (6), patient condition not explained (8), patient unlikely to improve (2), extended hospital stay (2), unexpected bill (2), doctor's rude behavior (2), poor attendance of doctor (2), lack of empathy (1), poor hospital administration (1), stress about patient condition (1).

Suggestions for improving Patient physician relationship from 142 attendants were sought and 170 responses were generated by the attendants which were grouped for analysis as shown in table 6. Attendants seek more doctor patient interaction, more time given to answer patient's queries, and that patients must be treated with love, care and compassion along with explanation of cost of services being provided.

Some of the responses about the factors responsible for WPV and suggestions of the attendants verbatim are:

- Incident occurred due to death of the patient. I became aggressive and verbally abused the doctor. I should have calmed down and not lost my control.
- Unexpected bill lead to physical confrontation. We were not allowed to see the patient for more than one hour in the morning and high cost medicines which were ordered were not used.
- Better education and awareness by government and big healthcare centres. Physicians should understand patient stress. Attendants should also not loose temper.
- Doctor should not scare the patient and treat the patient properly. Also money should not be the part of health review. Patient care should come first, then money.
- Doctors should tell the condition of the patient from time to time. Doctors should be more dedicated and attendants should also respect the doctor.

**Table 3:** Stress responses of healthcare workers on facing workplace violence

	*Not at all*	*A little bit*	*Moderately*	*Quite a bit*	*Extremely*
(a) Repeated, disturbing memories, thoughts or images of the abuse?	55	64	20	11	8
(b) Avoiding thinking about or talking about the abuse or avoiding having feelings related to it?	48	60	26	17	7
(c) Being “super-alert” or watchful and on guard?	16	26	37	40	42
(d) Feeling like everything you did was an effort?	31	33	26	47	21

**Table 4:** Perceptions of healthcare workers about the reasons that were responsible for workplace violence

*Responses*	*Physical violence* *N=11*	*Verbal abuse* *N=147*
Unexpected death	5(20)	46(12.5)
Unexpected complication	2(8)	49(13.4)
Patient condition not explained	3(12)	17(4.6)
Patient unlikely to improve	3(12)	52(14.1)
Extended hospital stay	3(12)	38(10.3)
Unexpected bill	1(4)	29(8)
Doctors’ rude behaviour	0	9(2.5)
Poor attendance of doctor	1(4)	14(3.8)
Staff shortage	4(16)	40(11)
Lack of empathy	1(4)	10(2.7)
Poor hospital administration	0	20(5.4)
Stress about Patient condition	1(4)	27(7.4)
Political links of patient	1(4)	16(4.3)
Total responses	25	367

Figures in parentheses indicate percentages

**Table 5:** Suggestions given by healthcare workers to improve patient physician relationship

*Administrative*	*Responses*	*HCWs*	*Responses*	*Patient/Relative*	*Responses*
Counseling sessions and counseling forms	26	Explain clinical condition and alan of care	40	Cooperate with doctor in decision making	21
Improve nurse patient ratio	23	Patient-healthcare worker communication improvement	33	Adhere to strict visiting hours	18
Improve doctor patient ratio	21	Proper counseling of relatives	31	Limited number of relatives	14
Adhere to strict visiting hours	18	Mutual respect and teamwork	23	Should trust the physician and healthcare workers	14
Strict and proactive security services	18	Explain expected cost and expected length of stay	12	Only the first degree relative should communicate with HCW about patient's condition	13
Grief counselor and financial counselor (health insurance)	15	Consultants available for counseling	10	Stay calm and focused	11
Minimize number of relatives	14	Protocols to minimize medical errors	4		
Strict action and law enforcement	9				
Video recording of counseling session	5				
Improve cost of services	5				
Limit paperwork	5				
Limit number of admissions	4				
Limit working hours of Junior Residents	4				
Total	167		153		91

## DISCUSSION

### Violence a Global Problem

Though violence against HCWs is a global problem, yet it is underreported and understudied.^1^ There has been rising incidence of violence against HCWs in developed as well as developing countries and the mutual trust between patient and physician has been broken.^3^ The World Health Organization (WHO) initiated a global campaign for violence prevention charting a plan of action for 2012-2020 asking the member nations to increase individual and institutional capacity for violence prevention and strengthen data collection and research on violence.^9^ News media, TV news and social media platforms are full of news in which the HCWs in hospitals are attacked mercilessly by the relatives of patients in case there is some complication, unexpected bills or demise of patient.^4–7^ It has been reported that in China mobs attack the hospitals and healthcare staff and number of Chinese school medical applicants have decreased.^10^ In a study among nurses, the annual incidence of verbal and physical assaults was 39% and 13% respectively whereas in another large study, 46% of nurses reported some type of WPV and one third among them were physically assaulted.^11,12^ A leading journal highlighted that the Chinese doctors are the victims of WPV and brought much needed global attention to this menace.^13^ In a multicentric study, 76% physicians in training reported verbal or physical violence during the previous 2 months whereas in another study it was reported that 74% of respondents were victims of violence during the preceding 12 months.^14^

**Table 6:** Suggestions given by the attendants to improve patient physician relationship

*Suggestions*	*Attendants’ Responses*
Treat the patient with love, care and politeness	77
More doctor patient interaction	32
More time to patient, answer queries, more frequent rounds	31
More respect to doctor and care for his/her job	13
Finances must be discussed/explain cost of treatment	8
Strict security (check for alcohol and other drug abuse)	4
More self control by patient's attendants	4
Diet and living arrangement of attendant	1
Total	170

### Perspective of HCWs

In the present study approximately 54% of HCWs had suffered workplace violence in the past one year which included physical assault (3.7%) as well. Maximum violence was faced by HCWs who were in the young age group and had less than 5 years of experience. As noted in other studies, it was observed that nurses, junior and senior residents are more prone to such episodes of violence as they are usually the frontrunners of a healthcare facility and are more in contact with patients and their attendants.^15^ A German study reported that almost 50% of practitioners were confronted with aggressive behavior, out of which 10% experienced critical to violent attacks, damage to property and/or physical assault.^16^ A study from India also reported that about 87% of violent incidents were verbal while 8.4% were physical.^17^

In this study it was also seen that higher number of incidents of WPV were reported during night shifts and in intensive care units (ICUs) and emergency than other areas. This implies that more security arrangements are required and more senior doctors need to be posted during night time in critical areas of hospital such as ICUs and emergency units. As was noted in our study, other studies have also implied that mostly patients themselves are not the troublemakers. It is usually the relatives and sometimes some unknown apparently sympathetic individuals or political leaders that may indulge in WPV.^18^ Another study also highlighted that 50% of the violent incidents occurred in the ICU and 70% of those were caused by relatives of patients.^3^ Unexpected death of the patient, unexpected complication and high cost of treatment were some of the factors responsible for WPV in our study as perceived by HCWs.

As shown in the study many HCWs reported moderate to high levels of stress and some of them had to take time off from duty. to cope with stress. Most of HCWs reported facing verbal abuse number of times in the past one year. It is evident that WPV creates a fearful work environment and also affects the decision making of doctors resulting in practice of defensive medicine. When these issues are not addressed in a timely and fair manner there is frustration, insecurity and resentment. Sometimes the HCWs conduct protests and demonstrations to make their voices heard.^19^

### Perspective of Attendants

Sixty two percent of attendants were aware of the fact that there was increasing incidence of violence against HCWs. Awareness was more in Govt and Private job holders, students and businessmen and it was higher in attendants who were educated up to high school, graduate and post graduate level. This shows a knowledge gap in the general population about rights and responsibilities of patient as well as healthcare worker. Twenty one attendants admitted to have been involved in physical and verbal confrontation with the HCWs in the past one year. In most cases the perpetrators were young and educated. The attendants who were involved in these incidents perceived that doctors’ behaviour was not up to the mark and they also reported dissatisfaction with the treatment. Most common reasons of WPV were unexpected complication in the patient, condition of patient not explained to the relatives along with high unexpected bill and doctor's rude behavior highlighting the importance of compassion, proper care, communication and counseling to the attendants.

Multiple factors have been attributed to rising incidence of healthcare related WPV in various studies. Long waiting time, delayed medical provision, patient dissatisfaction with nursing care, psychological stress and financial constraints were some of the possible causes of violence.^17^According to another study, main reasons of patient–physician distrust were patient perception of injustice, profit mongering, knowledge imbalances and physician conflicts of interest.^20^ The Lack of adequate training about communication, counseling and soft skills in dealing with patients in our medical curriculum may also be responsible for violent resolution of some conflicts.^21^

In the present times, healthcare delivery system is being equated to an industry and it is perceived to be working on same principles of profit and loss and the patient considers himself to be the consumer. General perception is that it is large profit-making enterprise and medical fraternity benefits monetarily, sometimes compromising on patient care and cost of treatment. Though some cases of unethical practices do happen as in any other profession also, but these facts are highlighted and exaggerated out of proportion by the media maligning the image of whole medical fraternity.^22^ The fact that people have to pay directly from their pocket as the health insurance cover is still poor in our country, cannot be undermined.^3^ So, when there is poor outcome or human loss along with financial constraints, there is emotional turmoil and even a small trigger act can result in aggressive behavior and violence.

### Bridging the Gap

Building a patient–physician relationship is essential for stress free work environment and better patient care. Some of the suggestions given by HCWs to decrease WPV were more counseling sessions, proper explanation of clinical condition and plan of care, improvement in patient-HCW communication, and information about the estimated cost of treatment and grief counselors who can support the attendants in case of adverse outcome. Highlights of suggestions given by attendants of patients were to treat the patient with love, care and politeness, more doctor patient interaction, dedicating more time to patient, answering queries and more frequent rounds could result in improved doctor patient interaction.

There must be a zero tolerance policy for abuse. Security must be improved especially in the night time and closed-circuit camera should be installed along with personal protection training of HCWs.^23^ Media, politicians and administration have to work optimistically rather than highlighting the rarest adverse outcome of medical practice and penalizing the HCWs who work for these sick patients in the best interest of public at large. Judicial system, law enforcement agencies, and government should also ensure that grievance of patients or the attendants as well as violence or aggression against HCWs is strongly dealt with in a fair manner.^3, 24^

Doctors’ protection Act 2010 states that any damage or act of violence against Medicare professionals is an act punishable by law and any damage to the property or the Institution of Medicare service is prohibited. Imprisonment to lawbreakers for a minimum period of 3 years and fine amount of INR 50,000 to be imposed if found guilty. Damage to any medical devices and equipment is a punishable offence and the offenders are liable to pay twice the amount of the damaged equipment's cost.^25^

## LIMITATIONS

The recollection of incidents of WPV by HCWs is memory based and may not be correct in some cases. As the incidents are mostly recollected from past experiences we could not directly analyze the factors responsible by asking the perpetrator, so it is the perception of HCWs that was recorded in responses. Though we did not study that if being in an act of violence changed the mindset and trust level of relatives towards HCWs and vice-versa forever or if it was an isolated incident. It being a cross sectional study another aspect which could not be evaluated; was aftermath or post-traumatic stress disorder in both HCWs and attendants.

## CONCLUSION

Studying the perspective of HCWs and attendants of patients, it is obvious that patient-physician relationship is fissured. This is going to impact the healthcare delivery in form of defensive medicine and high cost. There will be tendency of physicians not to get involved with critically ill patients where these incidents are rampant in current scenario. Much needs to be done to bridge this widening trust gap and the suggestions by HCWs and attendants highlighted the need of good communication, counseling, better patient care, decreased workload, empathy, compassion and improved cost of services. Security arrangements at the hospitals need to be given importance along with stringent law enforcement measures to create safe and fear free work environment of HCWs and a fair treatment to patients and attendants.
